# The ultra-short version of the Burnout Assessment Tool (BAT4)–development, validation, and measurement invariance across countries, age and gender

**DOI:** 10.1371/journal.pone.0297843

**Published:** 2024-02-23

**Authors:** Emina Hadžibajramović, Wilmar Schaufeli, Hans De Witte

**Affiliations:** 1 Institute of Stress Medicine, Region Västra Götaland, Gothenburg, Sweden; 2 School of Public Health and Community Medicine, Institute of Medicine, Sahlgrenska Academy at the University of Gothenburg, Gothenburg, Sweden; 3 O2L, Research group Work, Organizational and Personnel Psychology (WOPP), KU Leuven, Leuven, Belgium; 4 Department of Psychology, Utrecht University, Utrecht, Netherlands; 5 Optentia Research Unit, North-West University, Potchefstroom, South Africa; Universita Cattolica del Sacro Cuore Sede di Roma, ITALY

## Abstract

Given that burnout is a major problem in many societies and that employers are legally obliged to act in preventing job stress, there is a need of validated and reliable short self-report instruments. The Burnout Assessment Tool (BAT) is developed to measure burnout as a syndrome with four core components (exhaustion, mental distance, cognitive and emotional impairment). So far, the BAT was tested in over 40 studies with encouraging results. Although a short, 12-item version of the BAT exists, there is need for an ultra-short version with even less items. The overall aim is to develop an ultra-short 4-item version of the BAT (BAT4) and to evaluate its construct validity using Rasch analysis in samples from various countries along with its measurement invariance regarding country, age and gender. The BAT4 was developed using mixed methods, i.e. combining the results from a Rasch analysis, a subject matter analysis and expert judgements. Construct validity was tested on data from national representative samples from eight countries (the Netherlands, Belgium (Flanders), Austria, Czech Republic, Finland, Germany, Ireland, and Japan) and in a pooled sample combing the data from all eight countries. Differential item functioning regarding age, gender and country was investigated. The BAT4 fulfilled all the criteria required by the Rasch measurement model to constitute a valid measure in the pooled and country specific samples, except Austria and Japan. In the pooled sample, measurement invariance between the eight countries as well as between gender and age was found. Analyses within different countries showed occasional gender and age DIF for some items. The results were promising regarding BAT4’s construct validity and measurement invariance. Although the BAT4 includes only four items, its content coverage is acceptable. The BAT4 can be used as a short screening instrument for burnout complaints at the group or organisational level.

## Introduction

Burnout is a metaphor that refers to a state of work-related mental exhaustion. The notion of burnout was first introduced in the United States at the end of the 1970s [1], and to date it is recognized as an occupational disease or work-related disorder in several European countries [2]. Moreover, according to EU-regulations employers are obliged to periodically assess psychosocial risks among their employees and to implement policies to prevent job stress and burnout [3] (For the EU Occupation, Safety and Health Framework Directive see: https://osha.europa.eu/en/legislation/directives/the-osh-framework-directive/1). Hence the assessment of burnout with a validated, self-report inventory that can be included in a company’s psychosocial risk assessment is pivotal.

Currently, the Maslach Burnout Inventory (MBI) [4] is considered the gold standard to assess burnout as it has been used in about 90% of all studies on the subject [5]. The MBI includes three aspects of burnout: exhaustion, cynicism, and lack of professional efficacy. However, it has serious conceptual, psychometric, and practical flaws that have been discussed in more detail elsewhere [6]. For instance, on the conceptual level, serious doubts arose because rather than a constituting element of burnout, lack of professional efficacy seems to be a cause or consequence of burnout [7]. On the psychometric level it was shown that, for instance, reversing the positively worded professional efficacy items to indicate *diminished* professional efficacy, introduces an artefact as the correlations of the reversed positively worded efficacy scale are *much lower* than when a negatively worded scale is used [8]. Finally, the MBI was developed as a multi-dimensional research instrument and not as a practical assessment tool. Meaning that it measures the employee’s relation to his or her job–on each of the three dimensions–but does *not* produce an overall burnout score [9].

In order to overcome the conceptual, psychometric, and practical flaws of the MBI an alternative instrument was developed for individual and group-based assessment of burnout, the Burnout Assessment Tool (BAT) [6]. The BAT is based on the notion that burnout is a syndrome that includes: (1) exhaustion (i.e., a severe loss of energy that results in feelings of both physical and mental exhaustion); (2) mental distance (i.e., a strong reluctance or aversion to work, indifference, and cynicism); (3) cognitive impairment (i.e., memory problems, attention and concentration deficits, and poor cognitive performance); and (4) emotional impairment (i.e., intense emotional reactions such as anger or sadness, and feeling overwhelmed by one’s emotions). So, burnout is characterized by exhaustion and the concomitant reduced ability to regulate cognitive and emotional processes, as well as by mental distancing that acts as a counter-productive, ineffective coping strategy.

Originally, the BAT items were developed using Dutch language. Meanwhile, about 30 language versions are freely available (see: www.burnoutassessmenttool.be) and the BAT was used in over 40 studies with encouraging results. A recent overview [10] draws 5 major conclusions. (1) The underlying factorial structure of the BAT agrees with the notion of a burnout syndrome; implying that a unidimensional, total burnout score can be used, as well as 4 sub-scale scores. Moreover, this underlying bi-factor structure is invariant across countries, gender, age, and ethnicity. (2) The overall-BAT as well as its subscales are reliable in terms of internal consistency and stability across time. (3) Although the BAT is strongly correlated with other burnout measures, including the MBI, the overlap is far from complete, meaning that the BAT has distinctive features. (4) BAT-burnout can be distinguished from workaholism, job boredom, work engagement, anxiety, depressed mood, and general health. (5) The BAT fits well in the nomological network as described by the Job-Demands Resources model (Bakker & Demerouti, 2017). As postulated by this model, BAT-burnout mediates the relationship of job characteristics (demands and resources) and work outcomes, such as turnover intention, depression, and psychological distress. In sum, the BAT seems to be a valid and reliable instrument to assess the burnout syndrome.

Originally the BAT included 23 items [6] but by combining quantitative (Rasch analysis) and qualitative approaches (item content analysis and expert judgements) a shorter 12-item version of the BAT was developed [11]. Like the BAT23 [12] the BAT12 also fulfills the measurement criteria of the Rasch model, and works invariantly for older and younger employees, women and men and across two countries (i.e., The Netherlands and Flanders/Belgium). The previously mentioned review [10] also concludes that, generally speaking, research findings with the shortened 12-item version of the BAT are similar to that of the original 23-item version. This means that the short version of the BAT can be used without significant loss of information or precision. Yet, the BAT23 is preferred in clinical settings for more nuanced assessment using its 4 subscales which are–by definition–slightly more reliable than those of the shortened version.

Although a short, 12-item version of the BAT exists, there are practical advantages of developing an ultra-short version with even less items. In psychosocial risk surveys that many companies perform on a yearly basis or in large international surveys where working conditions across multiple countries are compared, many constructs are of interest to assess, such as various job demands, job and personal resources, leadership characteristics as well as health related outcomes such as burnout. Thus, surveys tend to be extensive, time-consuming and in a way mentally exhausting for employees to fill-in. Moreover, employers also have to balance the need of continuously monitoring the psychosocial work environment with the additional working hours needed to fill-in rather long surveys during work time. Thus, there are expectations on researchers to develop valid, reliable, yet short instruments without redundant items [13]. Concise and efficient scales including only core items can increase the probability of higher participation rates and also reduce participants fatigue and frustration [14].

Although there is a practical need for shorter instruments, their development and use impose some theoretical, statistical, and practical issues. From a psychometric point of view, removing items from psychometrically tested and in different contexts validated instruments is not recommended, as shortening instruments might result in poorer construct coverage and/or lower reliability [15]. As noted in the growing literature regarding scale-shortening strategies and consequences of their use, careful considerations are required in the development of short-scales and different strategies are available [16, 17]. Moreover, testing the different aspects of validity of the new version of an instrument is a neglected issue [18]. Following a similar strategy as for the development of the BAT12 [11], a mixed methods strategy will be applied in the development of the ultra-short version of the BAT–to include four items, one for each subscale. Theoretically and conceptually speaking, a 4-item version is considered the absolute minimum, aligning with the definition of the burnout syndrome according to the BAT, consisting of four primary symptoms. In order to increase the generalizability of the ultra-short BAT beyond the original Dutch version that is used in the Netherlands and Flanders, Belgium, we will analyse additional samples from six diverse countries, encompassing five different languages (Germany, Austria, Ireland, Finland, Czech Republic and Japan). This approach aims to address potential linguistic, cultural and regional differences.

Thus, the overall aim of the current paper is the development of an ultra-short 4-item version of the BAT–BAT4 –and to evaluate its construct validity using Rasch analysis in samples from various countries, along with its measurement invariance regarding country, age and gender.

## Methods

### The Burnout Assessment Tool

The shorter version of the BAT–BAT12 –was used as starting point for the development of the BAT4 [11]. The BAT12 includes twelve items, divided into four subscales (three items each): exhaustion (EX), mental distance (MD), cognitive impairment (CI), and emotional impairment (EI). All items are rated on a five-point frequency-based scale with response alternatives: never, rarely, sometimes, often, and always. The BAT12 fulfills the measurement criteria according to the Rasch model, can be considered as an unidimensional scale and works invariantly for different gender and age groups and between two countries (the Netherlands and Flanders, an autonomous region of Belgium) [11]. These results were replicated in a Romanian sample [19].

Previously, the BAT12 items have been analysed using a subject matter analysis [11, 20, 21] by which all items were classified into the following categories: a) *no problems with the item*, b) *wording errors*, c) *wording similar to one or more other items*, d) *item measures the same characteristic as one or more other items* and e) *item is an unclear measure of the construct*. The results showed that 10 out of 12 items were classified as *a) no problem with items*. Those ten items were: EX, MD and EI items, as well as the item CI5 (*I make mistakes in my work because I have my mind on other things*). Two remaining CI items, CI1 *(At work*, *I have trouble staying focused*) and CI4 (*When I’m working*, *I have trouble concentrating*) were classified as both a) no problems with item and d) items measure the same characteristic as one or more other items (To allow for comparison between different versions of the BAT, the numbering of the items refers to the BAT23.)

### Development of the BAT4

The shortening of the BAT12 to the BAT4 was done in a stepwise procedure. In the first step the BAT12 was reduced to BAT8 (one item from each subscale was deleted) and in the second step the BAT8 it was reduced to BAT4. This item reduction procedure is based on the logic that the BAT includes 4 symptoms, so the BAT8 includes 2 items per dimension, and the BAT4 only one item.

In the first step, reduction from 12 to 8 items was done by combining the results from the quantitative (item fit indicators from the Rasch analysis on the BAT12) and qualitative (subject matter analysis) analytic approaches. Items were chosen as candidates for deletion if misfit was indicated by a) one or more of the following item fit indicators: threshold ordering, items fit residuals, residual correlations and DIF regarding age, gender, and country; or by b) subject matter analysis. Item fit indicators are explained in the Rasch model section below. In addition to item fit indicators, graphical evaluation of item and threshold locations was done in an item-threshold map, to investigate the spread of the items across the latent trait. A similar item deletion strategy was used in the development of the BAT12 [11].

In the second step (shortening from 8 to 4 items), the decision about the item reduction could not be reached based on the item indicators nor subject matter analysis.

Hence, the decision to reduce the number of items from 8 to 4 was made through expert judgments, specifically, theoretical considerations of the item content conducted by the BAT developers (WS and HDW). The objective was to carefully choose the final four items from the remaining eight, ensuring comprehensive coverage of the core elements of each subscale and maintaining the requisite content validity for the ultra-short version of the BAT.

### Study population

The Dutch version of the BAT was used for the development of the BAT4 by using data from national representative samples in the Netherlands (NL: n = 1500; M_age_ = 42, Q1-Q3_age_ = 30–42; men = 54%, women = 46%) and Flanders, Belgium (FL: n = 1500; M_age_ = 41, Q1-Q3_age_ = 32–51; men = 54%, women = 46%) (M = median, Q1 = first quartile, Q3 = third quartile). Samples were representative for the Dutch and Flemish labor force regarding age, gender, and industry. Data was collected in the May-June of 2017 by a commercial survey agency (iVox). All information regarding the data collection can be found in a previously published study [6]. In the written informed consent, participants in the study were informed about the purpose of the study and its voluntary character and that they could stop at any moment. In the same information letter, contact details in case of questions or complaints were provided. Participants declared that they agreed with these terms by clicking on “next”. Ethical approval was received from the Social and Societal Ethics Committee of KU Leuven on June 16 2016, with a reference number: G-2016 06 2027 (https://www.kuleuven.be/english/research/ethics/committees/smec).

The Rasch analyses were performed using two subsamples of 800 participants, randomly chosen out of 2978 complete cases on all BAT items (NL = 1500, FL = 1478). The rationale behind this procedure was to check the robustness of the results by using two subsamples, which was possible due to the large sample size. Another reason was to enable more reliable differential item function analysis (DIF–explained below). If possible, it is recommended to include an equal number of participants within each group that is compared for DIF, to ensure that the larger group does not dominate the estimates of parameters [22]. The DIF was evaluated for gender (women/men), age (under/above median) and country (NL/FL). The two subsamples of 800 participants included randomly chosen 200 respondents each from the four strata: women/NL, men/NL, women/FL, men/FL. Age was split by median (under/over 41 years in each subsample). The random samples are drawn using the select random cases function in SPSS [23].

The construct validity of the BAT4 and DIF for gender and age within different countries was evaluated using national representative samples from the following countries: Austria (AU: n = 1054; M_age_ = 42, Q1-Q3_age_ = 29–53; men = 51%, women = 49%, data collected in December 2018), Germany (GE: n = 1073; M_age_ = 43, Q1-Q3_age_ = 30–55; men = 50%, women = 50%, data collected in December 2018), Finland (FI: n = 733; M_age_ = 42, Q1-Q3_age_ = 34–52; men = 50%, women = 50%, data collected in December 2019 to January 2020), Ireland (IR: n = 426; M_age_ = 41, Q1-Q3_age_ = 31–51; men = 54%, women = 46%, data collected in October 2017 to January 2018), Japan (JA: n = 1028; M_age_ = 39, Q1-Q3_age_ = 29–49; men = 50%, women = 50%, data collected in May 2018), Czech Republic (CZ: n = 964; M_age_ = 42, Q1-Q3_age_ = 32–51; men = 54%, women = 46%, data collected in September 2020, before the Covid-19 lockdown). All national samples were representative for age and gender. Representative samples from AU, GE, FI, FL, IR, JA and NL have been used previously in a study regarding the cross-national invariance of the long version of the BAT with 23 items [24]. More details regarding data collection in all countries except the Czech Republic can be found in the study by De Beer et al. [24]. Data from the Czech Republic was collected using the same procure. All data collections were done in accordance with ethical regulations in each country and included written informed consent For the purpose of this study, fully anonymized data file containing data from all countries was accessed on 10 January 2021.

The construct validity of the BAT4 was also tested in a pooled sample containing participants from each of the eight countries (n = 800). The pooled sample was used for the evaluation of the country DIF as well as for DIF regarding age and gender. For the above-mentioned reason regarding the need for an equal number of participants in groups when comparing for DIF, the country invariance of the BAT4 (DIF between countries) was evaluated in a combined dataset where 100 randomly selected participants from each of the eight countries were included (n = 800; 100 participant from each country: AU, CZ, FI, FL, GE, IR, JA, NL).

### The Rasch model and data analysis

Rasch analysis was used in the development of the BAT4, and for evaluation of its construct validity and measurement invariance regarding age, gender and country. The Rasch measurement model is a psychometric model for analyzing categorical data and represents the structure that data should show in order to obtain a measurement with good psychometric properties, and good construct validity. In contrast to the statistical modelling perspective, where the goal is to find the model that best characterizes a set of data, with the Rasch measurement paradigm, the goal is to evaluate whether a given set of data fits the requirement of the Rasch model [25]. Additionally, the emphasis is on identifying and studying possible anomalies in the data.

The requirements for a dataset to achieve fit to the Rasch model are unidimensionality, monotonicity, invariance, and local independency [26]. The Rasch model is a unidimensional latent trait model. Unidimensionality implies that all items should measure a common latent trait (burnout). Monotonicity is fulfilled if all items are positively related to that latent trait. The item responses need to be ordered in a probabilistic Gutman pattern [27] and this pattern needs to be observed across all items and response categories. In other words, persons with higher levels of burnout are expected to get higher scores on the BAT4 and vice versa. The invariance criterion means that the items need to work invariantly across the entire latent trait for all individuals. Furthermore, it is required that the items work invariantly (function in a similar way) for all comparable groups (e.g., between women and men, or between various countries), which is known as absence of differential functioning (DIF). The requirement of local independency implies that there should be no meaningful patterns among item residuals above and beyond the unidimensional latent trait of burnout. Local dependency can occur in the presence of multidimensionality (items measure more than a single latent trait) and/or response dependency among items [28]. Response dependency means that the two-item responses are linked in some way, i.e., answering one item in a certain way logically implies a specific response to other items. A pedagogical example of items that could cause response dependency are two inverse items included in the same instrument e.g. “*I feel alert*” and “*I feel tired*”.

In the present study, data was analyzed using RUMM2030 software [29] applying the partial credit model [30, 31]. The significance level was set at 0.01. The overall fit of the data to the Rasch model was evaluated by a χ2 statistic (expected to be non-significant, indicating invariance). Means and standard deviations for the overall person and item fit residuals were calculated with expected values around 0 and 1 respectively. The Person Separation Index (PSI) was used as measure of internal consistency and indicated the power of the scale to discriminate among respondents with different levels of burnout. The range of the PSI is 0 to 1, and its interpretation is similar to Cronbach’s alpha. To investigate unidimensionality, first a principal component analysis (PCA) on the item residuals was performed and items loading positively and negatively on the first principal component were used to obtain an independent person estimate. Independent t-tests of mean differences calculated for person estimates of positively and negatively loaded items were performed for each participant. According to the Smith’s test, unidimensionality is indicated if less than 5% of these differences are found outside the range of ±1.96 [32]. A 95% binomial confidence interval of proportions [33] was used to show whether the lower limit of the observed proportion is below the 5% level.

Besides the overall fit to the Rasch model, item fit indicators were also examined. *Threshold ordering* requires that response categories should be sequentially ordered across the latent trait for each item. *Item fit residuals* are expected to be within the range of ±2.5 with a non-significant χ2 statistic. *Residual correlations* between any two items are indicative of response dependence if their value is > 0.2 above the average correlation [34]. *DIF* was analyzed using ANOVA on standardized item residuals. The DIF analysis is based on the class interval which combines respondents with similar levels of burnout into distinct groups. The number of class intervals in the BAT12 analysis was ten, in the BAT8 it was 8 and in the BAT4 analysis it was six. As recommended by Andrich and Hagquist, the distinction between real and artificial DIF was investigated [22, 35, 36]. According to the recommended procedure, observed DIF was resolved sequentially, and new DIF-resolved analyses were performed, starting with the item with the highest F-value. The magnitude and impact of the DIF in the initial analysis was compared with the DIF-resolved analysis. In the DIF analyses, the significance level was Bonferroni adjusted and varies depending on the number of tests performed in each analysis. Post-hoc analysis for the DIF between countries was performed by pairwise comparisons using the Tukey’s HSD test. Moreover, DIF was also evaluated graphically by means of the item characteristic curves.

Finally, targeting of the BAT4 was evaluated graphically by the person-item-threshold plot. Targeting is understood as an aspect of what severity levels of burnout are covered by the items and whether they are in accordance with the participants levels of burnout. Targeting affects the precision of person estimates.

## Results

### The BAT4 development

#### Step 1 –BAT12 to BAT8

**Fig 1:**
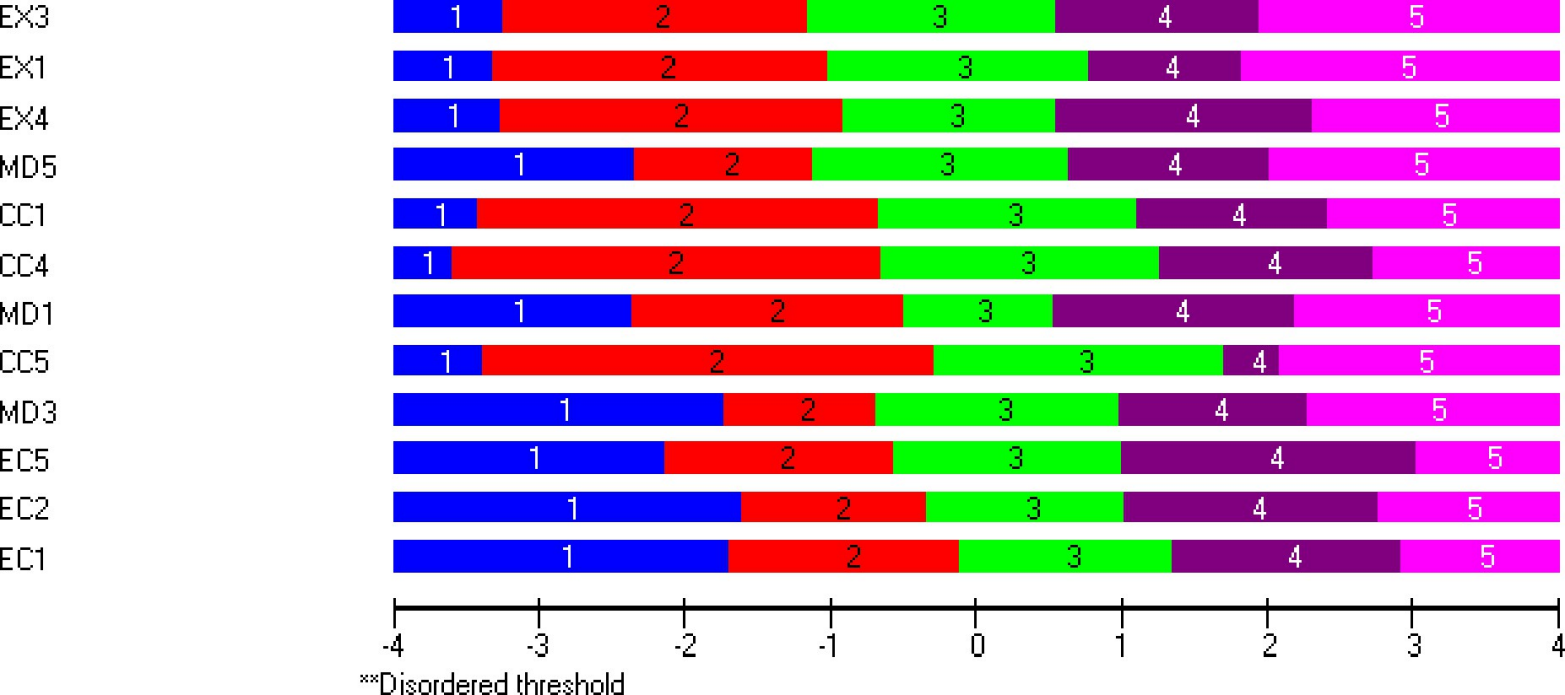
Item threshold map of the BAT12 items and thresholds ordering on a logit scale (higher value indicates higher burnout) of the Burnout Assessment Tool.

The Rasch model was fitted to the BAT12, and item fit indicators were scrutinised and shortly summarized here. Detailed information from this analysis was published in a previous study [11]. *Residual correlations*: In the BAT12 analysis, residual correlations above the value of 0.11 imply values that are higher than expected under the condition of local independence. Residual correlations above the critical value were observed for all pairs of items within the four subscales. The only exception was the pair MD3-MD5 for which the residual correlation was 0.11. Pairwise correlations for the four subscales were as follows: EX1-EX4 0.21, EX3-EX4 0.19 and EX1-EX3 0.16; MD1-MD3 0.28 and MD1-MD5 0.21; CI1-CI4 0.42, CI1-CI5 0.20 and CI4-CI5 0.27; EI1-EI2 0.34, EI1-EI5 0.36 and EI2-EI5 0.28. Concerning pairs of items belonging to different subscales, high residual correlations were not observed for any of the pairs. *Item fit residuals*: Item fit residuals outside the predefined range of ±2.5 were observed for the following items: EX3 (3.03), MD1 (-2.9), MD3 (-3.4) and EI5 (2.9). The χ2 values for item fit residuals were non-significant for all items. *DIF*: No DIF was observed for any of the EX, CI, or EI items. Gender DIF was observed for MD3. *Threshold ordering*: All 12 items had ordered thresholds which is visualised in Fig 1. At the bottom of Fig 1, the latent logit scale ranging from -4 to +4, indicates increasing levels of burnout from the left to the right. Looking at item threshold locations (positioning), the intended increasing levels of severity across the response categories (ranging from 1 = never to 5 = always) is reflected in the data for all items. *Item-threshold map*: In Fig 1, items are ordered in their locations (lowest to highest from top to bottom). The lowest locations were observed for the EX-items and are found at the top of the figure. This means that on average, the response shift from lower to higher responses, e.g., shift from 1 to 2 (*never* to *rarely*) or shift from 4 to 5 (*often* to *always*), was on average found at lower levels of burnout for the EX-items compared to MD and CI items.

*Item deletion*: Combining the results from the item fit indicators, item-threshold map, and subject matter analysis, one item from each subscale was chosen for deletion. *Exhaustion*: Item EX3 was chosen as candidate for deletion. The decision was made based on misfit on two indicators (residual correlations and item fit residual). Although item EX3 had the lowest location of all items (Fig 1), all three EX items were similar in terms of locations and thresholds patterns. *Mental distance*: Item MD3 was chosen as candidate for deletion. The decision to delete MD3 was based on misfit on multiple indicators (highest residual correlations among MD items, item fit residual and DIF regarding gender). Considering item locations in Fig 1, MD3 was the most difficult among the MD items and its location and threshold distribution along the latent trait was like the EI items. *Cognitive impairment*: According to statistical criteria CI1 and CI4 were possible candidates for deletion based on their high residual correlations. These items were also classified as possibly measuring the same characteristic in the subject matter analysis. Therefore, different combinations of BAT8 analyses were performed, deleting CI1 and keeping CI4 in the analysis, and the other way around, keeping CI1 and deleting CI4. *Emotional impairment*: Among the three EI items, item EI1 had the highest location of all items (Fig 1) and was retained to keep the spread of the items along the latent trait. Based on the pairwise residual correlations, both items EI2 and EI5 were possible candidates for deletion in the next step and were tested in different combinations of the BAT8 analyses.

Four different combinations of the BAT8 (BAT8a-d), including two items from each subscale, were evaluated regarding the overall model fit and the item fit statistics. Items EX1 and EX4 as well as items MD1 and MD5 were included in all BAT8 analyses. The following pairs of CI and EI items respectively, were evaluated in the different BAT8 models: CI1-CI5 (BAT8a, BAT8b) and CI4-CI5 (BAT8c, BAT8d), EI1-EI2 (BAT8a, BAT8c) and EI1-EI5 (BAT8b, BAT8d).

Overall model fit statistics for each model are shown in Table 1 and show that the best model fit according to the χ2 statistic was observed for the BAT8d. This was also the only model that did not indicate any DIF for gender, age, or country. Gender DIF was found for items MD1 and MD5 in the BAT8a and BAT8b analyses and item EX4 in the BAT8c analysis. In the four BAT8 models, item fit residuals outside the range of ±2.5 were noted for item EX4 (2.6 BAT8a), MD1 in all four analyses (-3.0, -3.3, -2.6 and 2.9, BAT8a through BAT8d) and EI2 (-2.7 BAT8b).

**Table 1:** Overall model fit statistics for random sample 1 and 2, n = 800 each.

	Item residual		Person residual		Chi square		Unidimensionality	
Analysis name	Mean	SD	Mean	SD	Value	P	PSI	Test % (95% CI)
BAT8a (random sample 1)	-0.21	1.90	-0.66	1.65	95.53	0.0008	0.87	7.1 (5.4;9.1)
BAT8b (random sample 1)	-0.12	2.06	-0.69	1.80	98.39	0.0004	0.86	7.1 (5.2;8.9)
BAT8c (random sample 1)	-0.23	1.73	-0.66	1.63	91.98	0.002	0.87	9.1 (7.2;11.3)
BAT8d (random sample 1)	-0.16	1.95	-0.68	1.76	82.43	0.01	0.86	7.4 (5.7;9.5)
BAT4 (random sample 1)	0.13	1.85	-0.59	1.36	34.66	0.02	0.75	3.5 (2.4;5.1)
BAT4 DIFsplit	-0.04	1.57	-0.58	1.34	33.26	0.12	0.75	
BAT4 (random sample 2)	0.37	0.48	-0.53	1.20	36.76	0.01	0.73	3.9 (2.7;5.6)

In Table 2, showing pairwise residual correlations for the BAT8 analyses, the highest correlations are found between the EI items. Between the two pairs of EI items (EI1-EI2 and EI2-EI5), the highest correlations were observed for the pair EI1-EI2 (Table2, analyses BAT8a and BAT8c). Thus, based on the overall model fit and the item fit statistics, the BAT8d was chosen for the next step (i.e., the decision was to delete items CI1 and EI1).

**Table 2:** Residual correlations for different combinations of BAT8 analyses.

	BAT8a	BAT8b	BAT8c	BAT8d
EX1-EX4	0.18	0.19	0.18	0.19
MD1-MD5	0.16	0.17	0.17	0.18
CI1-CI5	0.18	0.19		
CI4-CI5			0.25	0.25
EI1- EI2	0.34		0.33	
EI2-EI5		0.27		0.27

#### Step 2 –From BAT8 to BAT4

In the second step, the Rasch model was fitted to the remaining eight items: EX1, EX4, MD1, MD5, CI4, CI5 and EI2, EI5 (BAT8d analysis). All items had ordered thresholds, no DIF for gender, age or country was observed for any of the items and only item MD1 had an item fit residual outside the interval of ±2.5 (-2.9, non-significant χ2 statistic). Based on these results, it was not possible to eliminate items based on statistical criteria. Therefore, the final four items out of the eight remaining items, were chosen on theoretical reasoning.

Among the two EX items, EX1 (*At work*, *I feel mentally exhausted*) was chosen over EX4 (*At work*, *I feel physically exhausted*) because the former is more specific for burnout than the latter. For instance, physical exhaustion may also occur among those suffering from Chronic Fatigue Syndrome [37]. Additionally, mental exhaustion has always been at the core of the burnout syndrome [38].

Among the two MD items, MD1 (*I struggle to find any enthusiasm for my work*) was chosen over MD5 (*I’m cynical about what my work means to others*) because the former refers to a rather basic, ‘unfiltered’ emotion, whereas cynicism refers to a belief that results from cognitive appraisal. Item MD5 seems somewhat more specific, as it refers to others (and not everyone works with ‘others’ in their work), whereas enthusiasm in work seems something that can apply to everyone–hence MD1 has a broader ‘applicability’.

Among the two CI items, CI4 (*When I’m working*, *I have trouble concentrating*) was chosen over CI5 (*I make mistakes in my work because I have my mind on other things*) because concentration problems are supposed to happen more frequently than making mistakes. Besides, many mistakes are caused by poor concentration instead of the other way around, and having your mind on something else can also be due to other reasons than excessive workload (like boredom or external reasons, like poor health, conflicts outside work, etc).

Among the two EI items, EI5 (*At work I may overreact unintentionally*) was chosen over IE2 (*At work*, *I feel unable to control my emotions*) because overreacting unintentionally refers to overt behaviour and is therefore more concrete than not being able to control one’s emotions, which refers to covert behaviour and is therefore more abstract.

### The construct validity of the BAT4

The BAT4 included the following items: EX1 (*At work*, *I feel mentally exhausted*), MD1 (*I struggle to find any enthusiasm for my work*), CI4 (*When I’m working*, *I have trouble concentrating*) and EI5 (*At work*, *I may overact unintentionally*). Its construct validity was first evaluated using the data from NL and FL (random sample 1). The overall fit of the model was good according to the χ2 statistic (Table 1). The Smiths test for unidimensionality did not indicate any problem and the PSI was 0.75.

**Fig 2:**
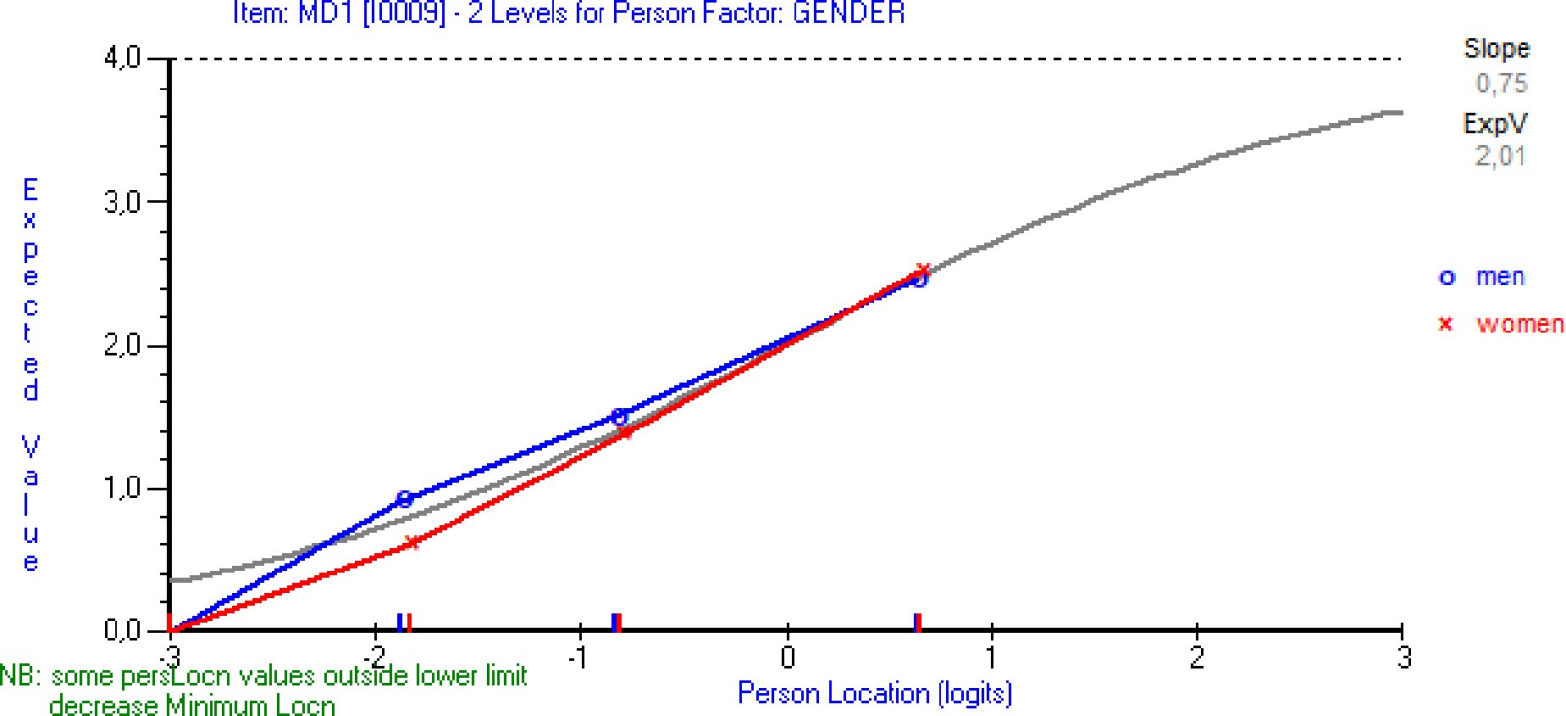
The item characteristic curve of item MD1 (I struggle to find any enthusiasm for my work)) for men and women.

Uniform DIF for gender was observed for item MD1 (F_1,716_ = 12.81, p = 0.0004). Additional analysis was done and item MD1 was split for gender (Table 2, BAT4 DIF split analysis). The difference between women’s and men’s location values for item MD1 was -0.25 logits with p-value = 0.01, which was the critical value for a significant difference. Differences between person mean values for women and men in the initial and the DIF resolved analyses were 0.34 and 0.24 logits respectively. The robustness of the results was tested in a second random sample (n = 800). DIF was not observed for any of the variables. In Fig 2 DIF was evaluated graphically and showed that men rated higher than women on this item given comparable levels of burnout, although the lines were close to each other. Therefore, it was concluded that adjustment for DIF was not justified. The overall fit to the Rasch model of subsample 2 is shown in Table 1 and was similar to the results of the subsample 1. The value of the χ2 statistic was 36.76 and the p-value was just at the significance level of 0.01. The Smith’s test indicated unidimensionality and the PSI value was 0.73.

Next, the BAT4 was tested using representative samples from six different countries, not included in the previous analysis: GE, AU, CZ, FI, IR, JA. The overall model fit for each country is shown in Table 3. According to the χ2 statistic, a good model fit was achieved for CZ, FI, GE and IR, whereas the p-value for AU and JA was below the cut-off value of 0.01. As can be seen in Table 3, according to the Smith’s test, the scale was unidimensional in each country. The value of the PSI value ranged from 0.64 (Ireland) to 0.77 (Japan).

**Table 3:** BAT4 –overall model fit statistics in different countries.

	Item residual		Person residual		Chi square		Unidimensionality	
Analysis name	Mean	SD	Mean	SD	Value	P	PSI	Test % (95% CI)
Austria	0.19	1.00	-0.36	0.95	43.76	0.002	0.70	3.2 (2.3;4.5)
Czech Republic	0.37	1.15	-0.40	1.07	20.13	0.45	0.67	4.1 (3.0;5.6)
Finland	0.24	1.05	-0.33	0.91	35.66	0.02	0.68	3.8 (2.6;5.5)
Germany	0.37	0.29	-0.40	1.01	33.95	0.03	0.71	1.5 (0.1;2.5)
Ireland	0.16	1.18	-0.36	0.94	27.69	0.12	0.64	2.2 (1.1;4.2)
Japan	0.28	1.74	-0,50	1.11	75.37	<0.0001	0.77	4.2 (3.1;5.7)
Pooled sample*	0.22	0.87	-0.42	1.04	25.91	0.17	0.72	1.8 (0.9;3.0)

*Randomly selected 100 participants from eight countries (AU, CZE, FI, FL, GE, IR, JA, NL)

### Differential item functioning regarding age and gender

The DIF analyses regarding gender and age for each country are summarized in Table 4 and visualised in S1 File. In the Irish and Finnish samples, no DIF was observed for any of the items. In the Austrian sample, significant DIF for age was observed for item CI4 (F_1,993_ = 16.68, p<0.0001, Bonferroni adjusted significance level was 0.0008). An additional analysis was done by separating this item for the two age groups. After splitting item CI4 for age, the overall χ2 statistic of the model fit was still significant (χ2 = 41.90, p = 002). The difference in item’s CI4 location for the two age groups (younger and older) was -0.29 logits with a significant p-value = 0.001. Differences between person’s mean values for younger and older respondents in the initial and the DIF resolved analyses changed only marginally, from 0.29 to 0.20 logits respectively, thus the DIF does not seem to have a substantial effect on the group averages. The same conclusion can be drawn from the graphical evaluation of the DIF as shown in Fig 1 found in S1 File.

**Table 4:** BAT4 ‐ differential item function regarding gender and age in different countries.

Country	Gender	Age
Austria		CI4
Czech Republic	EX1, MD1	EX1, MD1
Finland		
Germany	MD1	CI4
Ireland		
Japan	EX1	

**Fig 3:**
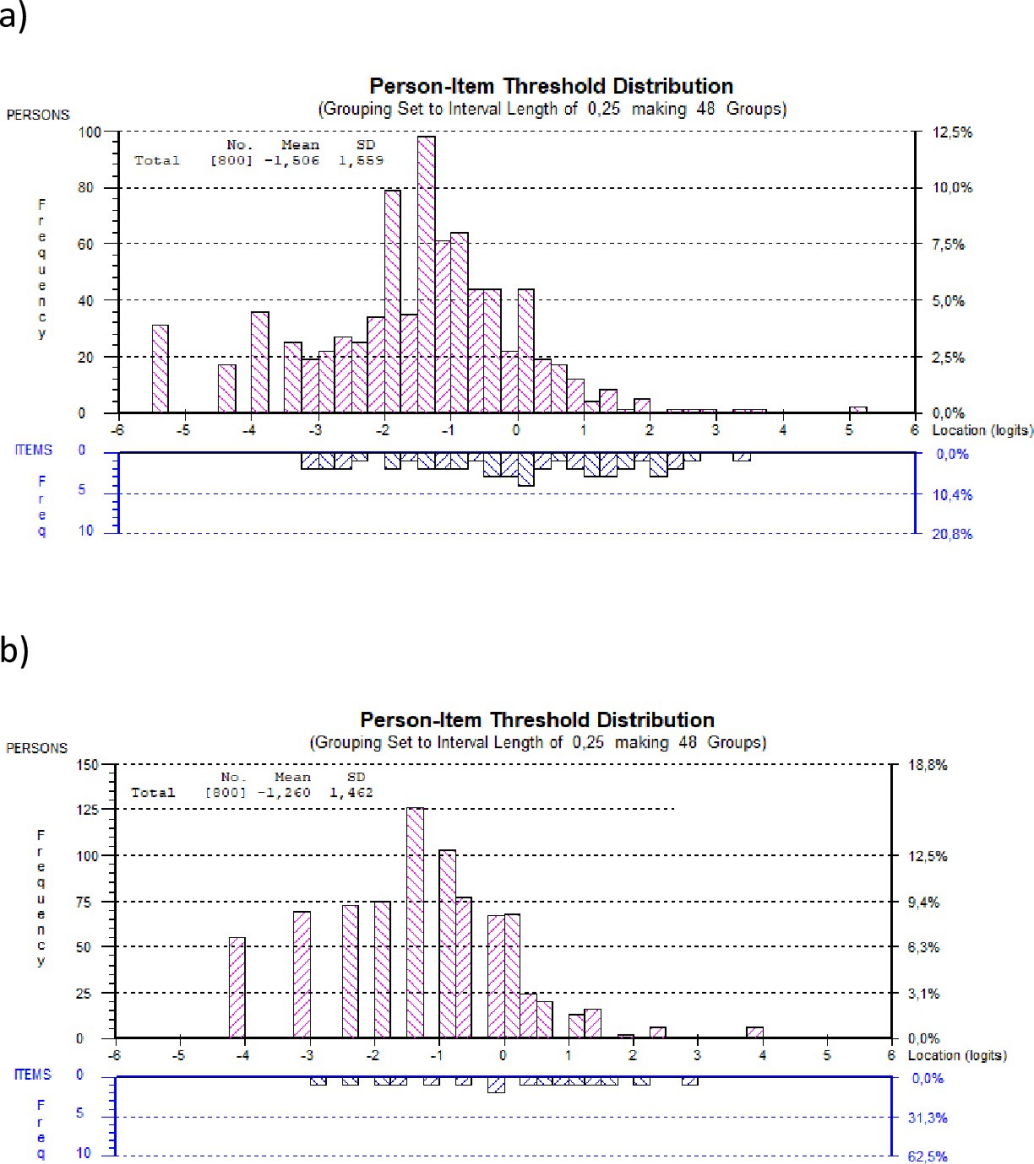
Person and item threshold distribution along the latent burnout scale (higher values indicate higher burnout levels) for: a) the BAT12 and b) the BAT4.

Item CI4 showed DIF for age in the German sample too (F_1,998_ = 29.36, p<0.0001, Bonferroni adjusted significance level was 0.0008). In both countries (AU and GE), given the same level of burnout, younger participants rated somewhat higher on item CI4 compared to older participants (see Figs 1 and 2 respectively found in S1 File). In the German sample, also DIF for gender for item MD1 was observed (F_1,998_ = 14.40, p = 0.0001, Bonferroni adjusted significance level was 0.0008; see also Fig 3 found in S1 File). The DIF issues were resolved sequentially in two additional analyses. In the first analysis, item CI4 was split for age (item with the highest F-value) and in that analysis, item MD1 still showed significant DIF for gender (F_1,998_ = 14.41, p = 0.0001, Bonferroni adjusted significance level was 0.0007). Thereafter, item MD1 was split for gender. In the second analysis, where both CI4 and MD1 were split for DIF, there was a significant difference in locations between younger and older respondents for item CI4 (diff = 0.516, p<0.0001) and between men’s and women’s locations for item MD1 (-0.412, p<0.0001). The value of the χ2 statistic was 45.96 with a p-value = 0.03. Once again, graphical evaluation of DIF revealed that the lines showing the estimates for different groups were close, thus the effect of DIF is questionable (Figs 2 and 3 found in S1 File).

In the Czech sample both age and gender DIF was noted for items EX1 (age: F_1,925_ = 12.68, p = 0.0004; gender: F_1,925_ = 12.60, p = 0.0004, Bonferroni adjusted significance level was 0.0008) and MD1 (age: F_1,925_ = 14.53, p = 0.0002; gender: F_1,925_ = 18.58, p<0.0001, Bonferroni adjusted significance level was 0.0008). Next, item MD1 was split by gender (item with highest F-value), which resulted in the disappearance of the gender DIF for item EX1 (F_1,925_ = 3.53, p = 0.06, Bonferroni adjusted significance level was 0.0007) and the disappearance of the age DIF for item MD1 (women: F_1,925_ = 4.43 p = 0.04; men: F_1,925_ = 11.07, p = 0.0009, Bonferroni adjusted significance level was 0.0007). The DIF for age regarding item EX1 was still present (F_1,925_ = 12.50, p = 0.0004, Bonferroni adjusted significance level was 0.0008). Consequently, an additional analysis was executed to resolve the age DIF for item EX1. In that analysis, age DIF for item MD1 disappeared (F_1,925_ = 5.39, p = 0.02) as well as the gender DIF for item EX1 (younger: F_1,925_ = 3.94 p = 0.05; older: F_1,925_ = 8.60, p = 0.004, Bonferroni adjusted significance level was 0.0007). Gender DIF for MD1 was still significant (F_1,925_ = 13.57, p<0.0001). We can conclude that the DIF for these items could be spurious (artificial DIF). A graphical presentation of DIF is found in Figs 4–7 in S1 File.

Finally, in the Japanese sample gender DIF was observed only for item EX1 (F_1,975_ = 20.35, p<0.0001). Consequently, item EX1 was separated for women and men. In the DIF resolved analysis, the difference in locations between women and men for item EX1 was 0.49 logits, which turned out to be not statistically significant (p = 0.57). Differences between person mean values for men and women in the initial and the DIF resolved analyses were 0.01 and 0.20 respectively. A graphical evaluation of the DIF is shown in Fig 8 found in S1 File. The fit to the Rasch model was not improved in the DIF resolved analysis (χ2 = 75.19, p<0.0001).

### Measurement invariance between different countries

The BAT4’s measurement invariance between eight countries (NL, FL, GE, AU, CZE, FI, IR, JA) was evaluated by means of DIF analysis performed on a pooled sample containing 100 participants from each country. In the same model, DIF for gender and age was also evaluated. The overall model fit is presented in Table 3 and the data coincided with the Rasch models expectations. DIF for country was noted for item CI4 (F_1,738_ = 4.66, p<0.0001, Bonferroni adjusted significance level was 0.0008) (see also Fig 9 in S1 File). Post hoc analysis showed that significant differences were found between FL and JA (Diff = 0.48 p = 006) and between FL and CZE (Diff = 0.64 p-value <0.001). The same item also showed DIF for age (F_1,738_ = 16.72, p<0.0001) and is shown in Fig 10 in S1 File. In the next step, the DIF age was resolved for CI4 (item with the highest F-value). In the analysis where the CI4 was split by median age into younger and older participants, the country DIF for the same item disappeared (younger F_1,738_ = 2.28, p = 0.03, older F_1,738_ = 2.91 p = 0.006, Bonferroni adjusted significance value was 0.0007) which implied artificial DIF and consequently suggested country invariance of BAT4.

Finally, the distribution of the study participants and the BAT12 and the BAT4 item thresholds along the latent burnout scale is shown in Fig 3 (targeting). Although the number of items was reduced to only 4 and consequently, there are some gaps between the item thresholds (the lower part of the figure), the spread of the items of the ultra-short scale across the latent trait is similar to that of the longer version. In both graphs, there is a group of participants with burnout levels that are lower than the level covered by the items, which implies that the precision in estimates for these participants is less precise. The same information can also be noted in the overall sample mean, -1.260 (SD = 1.462) for the BAT4, compared to the item mean, which is constrained to 0. Thus, the targeting of the BAT4 is not optimal (especially for participants with very low levels of burnout) but can still be considered as acceptable.

## Discussion

The overall aim of the present study was to develop an ultra-short version of the BAT, which was achieved using mixed methods, i.e., combining the results from a Rasch analysis, a subject matter analysis and expert judgements. An additional aim was to evaluate the BAT4’s construct validity and measurement invariance in national representative samples from eight different countries. In six out of eight countries (NL, FL, CZ, GE, FI and IR) the BAT4 fulfilled all the criteria required by the Rasch measurement model to constitute construct validity of a scale. In the Austrian and Japanese samples, the fit to the Rasch model was not achieved. Good fit to the Rasch measurement model was also achieved when participants from eight countries were combined in a pooled sample. In the pooled sample, measurement invariance between the eight countries as well as between gender and age was found.

In the Austrian and Japanese samples, the chi-square statistics indicated misfit to the Rasch model. These results should be interpreted with caution, as inference based on chi-square statistics is sensitive to large sample sizes which can produce bias. The sample size in both countries was >1000, which could imply that even minor levels of misfit become statistically significant [39]. Analyses within different countries showed occasional gender DIF for items EX1 (CZ and JA) and MD1 (CZ, GE and NL/FL); and age DIF for items EX1 (CZ), MD1 (CZE) and CI4 (AU, GE). One avenue for further research could be to evaluate whether these results persist when analyses are repeated on new samples. In addition to the statistical evidence of invariance, another important aspect when self-rated instruments are to be used across countries, is to make sure that items are translated well linguistically and also culturally adapted to preserve content validity at a conceptual level across various cultures [40]. Problems with language/translation in the Austrian sample can perhaps be ruled out, because the same German version was used in both Germany and Austria. As far as the Japanese sample is concerned, cultural/language factors might play a role. On the other hand, in a study by Sakakibara et al, a careful translation and cultural adaptation process of the Japanese version of the BAT (long version) was reported and good factorial and construct validity was found [41]. Moreover, measurement invariance between seven countries (including both Austrian and Japanese data) when using the long version of the BAT was found in a study by De Beer et al. [24].

The shortening of an instrument has an effect on the content coverage i.e. the construct validity as well as on their precision on a group and individual level [42]. This implies that shorter instruments have lower power to detect change and/or discriminate between individuals. The scale’s length has an effect on the score’s distribution (increased risk of floor and ceiling effects) and correlations with other constructs i.e., the scale’s predictive validity, concurrent and discriminant validity can be affected. One advantage of the present study is that the theoretical reasoning of the content of the items was incorporated along with the results from the statistical analyses. Theoretical reasoning implies that the experts in the filed reach a consensus about which items best cover the construct of interest [43]. Relying solely on statistical results and using methods belonging to classical test theory, often results in choosing items with the aim to maximize the reliability coefficient of internal consistency (usually Cronbach’s alpha) [42, 43]. Although this strategy is straightforward and makes sense in a classical test theory paradigm, one consequence is that the items are selected based on their highest correlation with other items. A limitation of this approach is that highly correlated items tend to be similar, which in turn narrows the construct coverage.

In the present study, combining qualitative and quantitative methods, the spread of the items across the latent trait seems to be preserved. The targeting of the BAT4 items was compared with those of the BAT12. Although the spread of the items across the burnout latent trait was not optimal, especially for individuals with very low burnout levels, the targeting of individuals with higher levels of burnout was acceptable. This targeting was interpreted as acceptable based on the reasoning that individuals with very low burnout levels are probably the healthiest participants and those are usually not the target groups of workplace interventions. Workplace interventions are often tailored to cope with high levels of burnout which are associated with increased risk for negative health consequences. The PSI value for the BAT4 ranged between 0.64 and 0.77 in different countries and was 0.72 in the pooled sample. These levels can be considered as high enough to at least allow comparison of participants on the group level. Thus, we believe that the BAT4 could be a useful measure for screening purposes (group or organisational levels of burnout–however not as a diagnostic tool) to get a first rough identification of persons at risk for burnout. In clinical settings, the long version of the BAT should be used (BAT23) as a diagnostic aid, preferably also with addition of the secondary symptoms. Please note that for a diagnosis, more information is needed through a clinical interview, including an anamnesis.

We believe that the BAT4 holds potential as an effective solution for conducting extended, large-scale epidemiological studies aiming to assess a significant number of relevant concepts. Such large-scale surveys obviously need very short scales, that are still valid and reliable. One example is the ultra-short 3-items version of the Utrecht Work Engagement Scale that has been added to the European Working Condition Survey, as the previous 9-items version was too long to be included. For users of the ultra-short version of the BAT, as highlighted in a previous publication regarding cut-off points for the long (BAT23) and shorter (BAT12) version of the BAT [44], we recommend that aggregated group levels can be reported to organisations, whereas individual scores should only be disclosed to individual participants.

Another strength of this study is that the development of the BAT4 was done using representative samples of the Dutch version of the BAT. Later on, its construct validity was tested on large representative samples from various counties as well as on the pooled sample from all countries. Large samples can also be a limitation due to the fact that even minor deviations become statistically significant. In the development of the BAT4 using data from NL/FL, we have tried to address this issue by randomly selecting a smaller sample size and by repeating the construct validity analysis in a new randomly selected sample of the same size (n = 800). The construct validity of BAT4 and its measurement invariance between the eight countries, age and gender is further tested in a pooled sample using data from all eight countries, as well as within each country.

Although the results of this study regarding the BAT4’s construct validity and measurement invariance regarding age, gender and across countries seems promising, it should be emphasized that this is its first evaluation, and additional research is required, i.e., testing the psychometric properties of the BAT4 with other analytic methods and/or other data. In this study, the BAT4 was evaluated across seven European countries and in Japan. Additional validation utilizing data from diverse geographical regions and continents is recommended for a comprehensive evaluation of its applicability. Moreover, another avenue for future research should focus on the usability of the BAT4 such as its correlation with other burnout measures (convergent validity), its relationship with other constructs such as workaholism, job boredom, work engagement(discriminant validity) and its predictive validity related to organizational and health outcomes, such as work performance and sickness absence. Future studies should also evaluate whether cut-off scores for the BAT4 can be developed in an analogous way as was done for the BAT23 and BAT12 [44].

## Conclusion

In six out of eight countries, as well as in the pooled sample, the ultra-short version of the BAT (BAT4) fulfils the criteria required by the Rasch measurement model to constitute a valid and reliable scale for the assessment of burnout complaints. In a pooled sample, the BAT4 works invariantly between the eight countries, gender and age. In country specific analyses, some minor age and gender differences were observed that should be further investigated in new samples and/or by using different analytical methods.

The results were promising regarding BAT4’s construct validity and measurement invariance. Although the ultra-short version includes only four items, its content coverage is acceptable. Further studies are needed to evaluate its practical usability. The BAT4 can be used as a short screening instrument for burnout complaints at the group or organisational level.

## Supporting information

S1 File Differential item functioning graphs. (DOCX)

S2 File Data material. (XLSX)
