# Supplementary material for: The ultra-short version of the Burnout Assessment Tool (BAT4)–development, validation, and measurement invariance across countries, age and gender
Source: PLoS One. 2024 Feb 23;19(2):e0297843. doi: 10.1371/journal.pone.0297843 (PMC10889892; doi:10.1371/journal.pone.0297843)
Supplement: S1 File — (DOCX) [file pone.0297843.s001.docx]

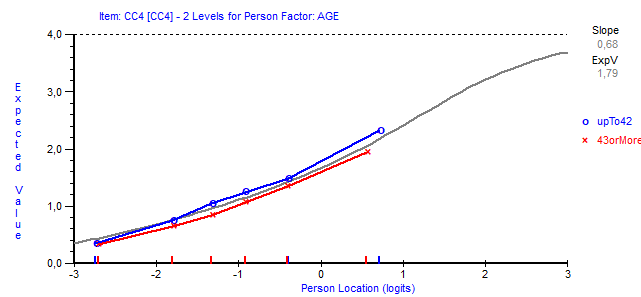


**Fig 1. The item characteristic curve of item CI4 (*When I’m working, I have trouble concentrating*) for younger (up to median age of 42) and older (over median age of 42) participants in a sample from Austria (n=1054).**


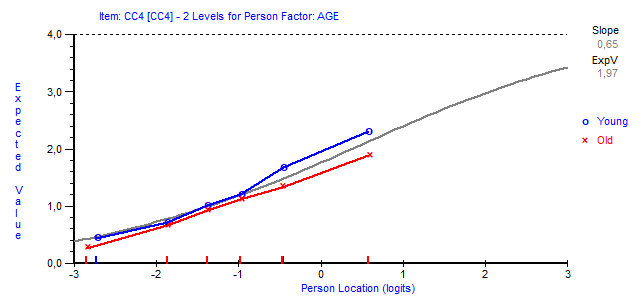


**Fig 2. The item characteristic curve of item CI4 (*When I’m working, I have trouble concentrating*) for younger (up to median age of 42) and older (over median age of 42) participants in a sample from Germany (n=1073).**


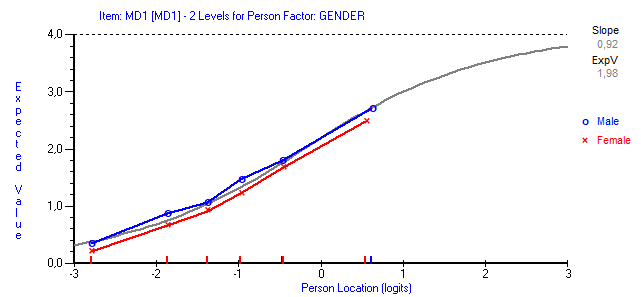


**Fig 3. The item characteristic curve of item MD1 (I struggle to find any enthusiasm for my work) for women and men in a sample from Germany (n=1073).**


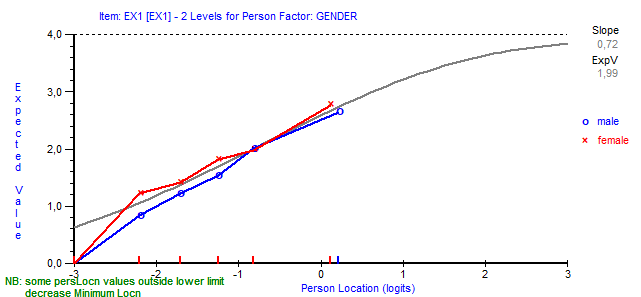


**Fig 4. The item characteristic curve of item EX1 (*At work, I feel mentally exhausted*) for women and men in a sample from Check Republic (n=964).**


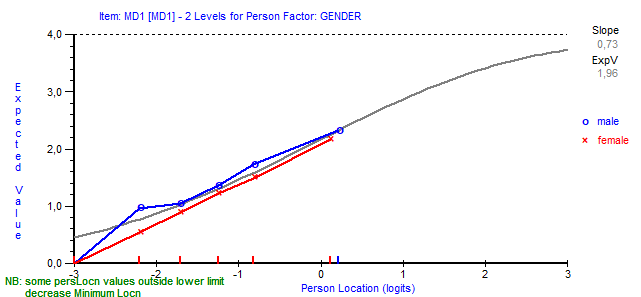


**Fig 5. The item characteristic curve of item MD1 (I struggle to find any enthusiasm for my work) for women and men in a sample from Czech Republic (n=964).**


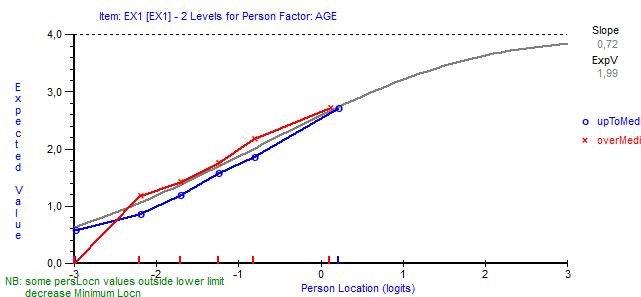


**Fig 6. The item characteristic curve of item EX1 (*At work, I feel mentally exhausted*) for younger (up to median age of 42) and older (over median age of 42) participants in a sample from Check Republic (n=964).**


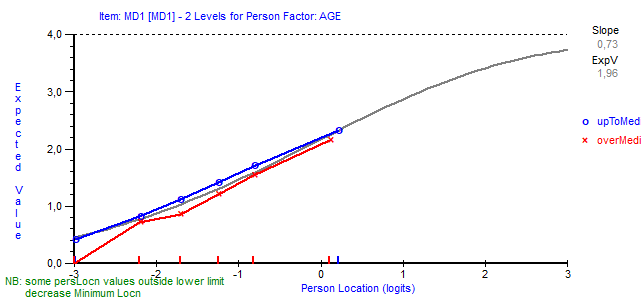


**Fig 7. The item characteristic curve of item MD1 (I struggle to find any enthusiasm for my work) for younger (up to median age of 42) and older (over median age of 42) participants in a sample from Czech Republic (n=964).**


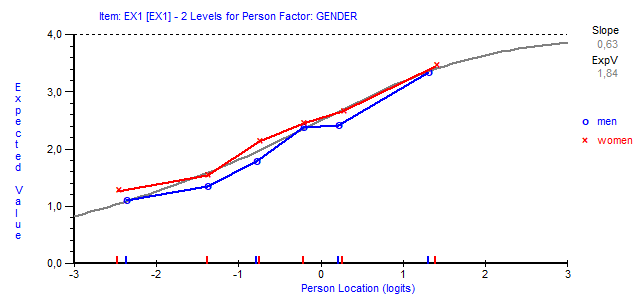


**Fig 8. The item characteristic curve of item EX1 (*At work, I feel mentally exhausted*) for women and men in a sample from Japan (n=1028).**


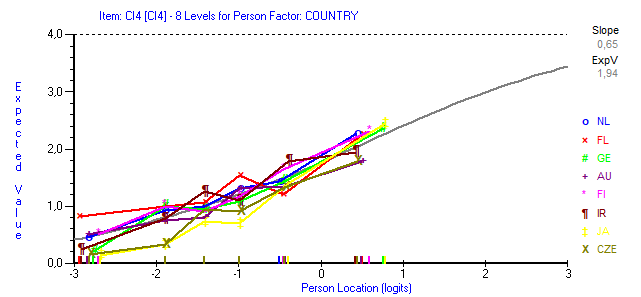


**Fig 9. The item characteristic curve of item CI4 (*When I’m working, I have trouble concentrating*) for participants in different countries (NL= Netherlands, FL= Belgium, Flanders, GE=Germany, AU=Austria, FI=Finland, IR=Ireland, JA=Japan, CZE= Czech Republic) in a sample including 100 randomly selected participant from each country (n=800).**


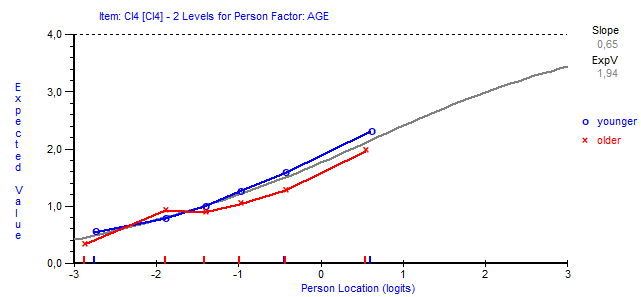


**Fig 10. The item characteristic curve of item CI4 (*When I’m working, I have trouble concentrating*) younger (under the median age) and older (above the median age) participants in a sample including 100 randomly selected participant from following countries Netherlands, Belgium, Flanders, Germany, Austria, Finland, Ireland, Japan, Czech Republic) (n=800).**
